# Impact of the roll out of comprehensive emergency obstetric care on institutional birth rate in rural Nepal

**DOI:** 10.1186/s12884-017-1267-y

**Published:** 2017-03-04

**Authors:** Sheela Maru, Alex Harsha Bangura, Pooja Mehta, Deepak Bista, Lynn Borgatta, Sami Pande, David Citrin, Sumesh Khanal, Amrit Banstola, Duncan Maru

**Affiliations:** 1 Possible, Bayalpata Hospital, Sanfebagar-10, Achham, Nepal; 2 Department of Obstetrics and Gynecology, Boston Medical Center, Boston, MA USA; 3 Department of Obstetrics and Gynecology, Boston University School of Medicine, Boston, MA USA; 4 Department Medicine, Division of Women’s Health, Brigham and Women’s Hospital, Boston, MA USA; 5 Contra Costa Regional Medical Center, Contra Costa Family Medicine Residency, Martinez, CA USA; 6 United Nations Population Fund, Kathmandu, Nepal; 7 Department of Anthropology, University of Washington, Seattle, WA USA; 8 Department of Global Health, University of Washington, Seattle, WA USA; 9 Henry M. Jackson School of International Studies, University of Washington, Seattle, WA USA; 10 Institute of Medicine, Tribhuvan University, Kathmandu, Nepal; 11 Faculty of Health and Applied Science, University of West England, Bristol, UK; 12 Public Health Perspective Nepal, Department of Research and Training, Pokhara, Nepal; 13 Department of Medicine, Division of Global Health Equity, Brigham and Women’s Hospital, 75 Francis Street, Boston, MA USA; 14 Boston Children’s Hospital, Department of Medicine, Division of General Pediatrics, Boston, MA USA; 15 Departments of Medicine and Global Health and Social Medicine, Harvard Medical School, Boston, MA USA

**Keywords:** Maternal mortality, Institutional birth rate, Global health, Implementation research, Nepal

## Abstract

**Background:**

Increasing institutional births rates and improving access to comprehensive emergency obstetric care are central strategies for reducing maternal and neonatal deaths globally. While some studies show women consider service availability when determining where to deliver, the dynamics of how and why institutional birth rates change as comprehensive emergency obstetric care availability increases are unclear.

**Methods:**

In this pre-post intervention study, we surveyed two exhaustive samples of postpartum women before and after comprehensive emergency obstetric care implementation at a hospital in rural Nepal. We developed a logistic regression model of institutional birth factors through manual backward selection of all significant covariates within and across periods. Qualitatively, we analyzed birth stories through immersion crystallization.

**Results:**

Institutional birth rates increased after comprehensive emergency obstetric care implementation (from 30 to 77%, OR 7.7) at both hospital (OR 2.5) and low-level facilities (OR 4.6, *p* < 0.01 for all). The logistic regression indicated that comprehensive emergency obstetric care availability (OR 5.6), belief that the hospital is the safest birth location (OR 44.8), safety prioritization in decision-making (OR 7.7), and higher income (OR 1.1) predict institutional birth (p ≤ 0.01 for all). Qualitative analysis revealed comprehensive emergency obstetric care awareness, increased social expectation for institutional birth, and birth planning as important factors.

**Conclusion:**

Comprehensive emergency obstetric care expansion appears to have generated significant demand for institutional births through increased safety perceptions and birth planning. Increasing comprehensive emergency obstetric care availability increases birth safety, but it may also be a mechanism for increasing the institutional birth rate in areas of under-utilization.

## Background

The greatest lifetime risk for a mother and her baby occurs during childbirth; over 800 women die from preventable childbirth-related causes every day [1]. More than 40% of the world’s 535,900 annual maternal deaths are related to intrapartum complications, which are closely linked to the world’s two million annual intrapartum and neonatal deaths [2, 3]. 99% of these deaths occur in low- and middle-income countries [1].

Increasing the institutional birth rate (IBR) is a central strategy in reducing mortality, yet several factors continue to challenge progress. Nepal, one of South Asia’s most impoverished countries, is a paradigmatic case of these challenges. In 2011, the maternal mortality ratio in Nepal was estimated at 281 per 100,000, and only 35% of births took place in a healthcare facility [4].

The Three Delays Model offers a useful framework for understanding the barriers to achieving institutional birth: the first delay occurs with care-seeking, the second in arrival at a healthcare facility, and the third in the provision of appropriate care [5]. The first and second delays are often considered demand problems. Maternal age, parity, education, and household wealth have all been positively associated with service usage [6]. These factors suggest that experience with labor, awareness of danger signs, autonomy, and financial support all increase demand for maternal healthcare. Government-supported financial incentives and outreach programs have been in place in Nepal since 2009, and are attributed to increased institutional birth [7]. However, rural Nepal is a patriarchal society where a woman often must defer healthcare decisions to her husband or his family members, particularly the mother-in-law [8]. Lack of support from this older generation remains a key barrier to maternal healthcare services utilization [8–11], and may be more important than cost or lack of awareness [9]. While financial, educational, and participatory action-based community mobilization strategies have shown success in improving utilization in Nepal and elsewhere, there is limited evidence that increased demand alone improves maternal and neonatal outcomes [12, 13].

The third delay, the provision of appropriate care, is a supply problem addressed by increasing availability of trained providers and well-equipped facilities critical to improving outcomes [13]. To significantly reduce maternal mortality, the World Health Organization recommends at least four basic emergency obstetric care (BEmOC) facilities and one comprehensive emergency obstetric care (CEmOC) facility per 500,000 people [14]. CEmOC includes cesarean section, blood transfusion services, and sick newborn care in addition to all five signal BEmOC functions (parenteral antibiotics, uterotonics, and anticonvulsants; manual placenta removal; removal of retained products of conception; assisted vaginal delivery; and basic newborn resuscitation) [14]. While evidence for impact of intrapartum interventions and EmOC on maternal outcomes remains inadequate in low-resource settings [15, 16], it is estimated that universal CEmOC coverage would avert 519,000, or 85% of intrapartum-related neonatal deaths per year [17].

The three delays are interrelated, as decisions about when and where to seek care are limited by available services. Others have found that increasing emergency obstetric services leads to increased institutional birth and that women consider service availability when deciding where to deliver [18–20]. Importantly, a recent quasi-experimental study comparing demand- and supply-side interventions demonstrated that when financial incentive programs were followed by increased access to BEmOC, communities saw the greatest gains in institutional birth, particularly for the most impoverished groups [21]. However, the dynamics of how maternal healthcare-seeking behavior evolves during CEmOC expansion have not been studied. In this study, we explore obstetric healthcare-seeking behavior for women living in the catchment area population of a facility undergoing transition from BEmOC to CEmOC. We examine barriers to care, as understood through the Three Delays Model, prior to and after this transition. Understanding these barriers and factors in women’s decisions has important implications for quality improvement, health education, and outreach.

## Methods

We conducted this research in collaboration with the non-profit organization *Possible*, which operates Bayalpata Hospital as a public-private partnership with the Ministry of Health of Nepal. Bayalpata Hospital provides free care to a catchment area population of approximately 36,000 people in 14 village clusters in Achham, one of the poorer districts in Nepal’s hilly Far-Western Development Region [4]. Over the course of the study, the hospital expanded its services from BEmOC to CEmOC. In a district of approximately 260,000 people, there is only one other physician-staffed facility, and six of the area’s 14 village clinics (known in Nepal as health posts) were designated as BEmOC facilities during the study.

For this pre- and post-intervention cohort study on the impact of CEmOC expansion on institutional birth in rural Nepal, we surveyed an exhaustive sample of all women less than six weeks postpartum during three month periods before and after CEmOC expansion. The sample included two populations of postpartum women who: (1) delivered in the community (at home or at a clinic) and (2) either delivered or received services for postpartum complications in the hospital. Women presenting to the hospital were identified, consented, and interviewed by nurse-midwives, while women delivering in the community were identified by government community health workers (CHWs, known locally as Female Community Health Volunteers) and interviewed by the hospital’s paid CHWs.

All postpartum women within six weeks of birth and living in the catchment area were eligible for participation in the study. Women referred to another facility after arriving at Bayalpata Hospital were later excluded due to difficulty in determining outcomes in this dispersed population. There were no other exclusion criteria. Women received NRs 100 (approximately 1 USD) compensation for participating. Government CHWs received NRs 50 for each woman identified and nurse-midwives received NRs 100 for each survey administered. Bayalpata CHWs received no additional compensation, as these activities are part of their employment contract.

In addition to demographic questions, we asked the women about their choice of delivery location, their beliefs around safe delivery practices, the factors important in their decision-making process, and their satisfaction with their choice. We elicited factors through open-ended questioning and later coded them into themes; dummy variables were then used to enumerate the frequency of each factor within and between groups. All other questions were multiple choice.

We coded all quantitative data on paper and manually entered the codes into an excel spreadsheet. Identifiers were not collected. We coded and analyzed the pre-intervention data separately for a previous study on drivers of institutional birth [22]. After completing the post-intervention surveys, we added the data to a pooled dataset and re-analyzed them using JMP version 11 (SAS Institute Inc., Cary, NC 2013). We conducted bivariate analysis of demographics across periods and factors associated with institutional birth, and within and across periods. We used Fisher’s exact test for categorical variables (e.g., caste, parity, delivery location, priority factors), and the Wilcoxon rank-sum test for all continuous variables (e.g., age, income, land, distance, travel cost), as they had non-normal distributions. We determined significance with an alpha-cutoff of 0.05. For the logistic regression analysis, we entered all significant variables in the bivariate analyses (listed in Tables 1 and 2) into the model and refined using manual backward elimination, with an alpha cut-off of 0.05. Those variables that did not meet the cut-off are not reported in the final model.

**Table 1:** Sample characteristics and demographics

Sample demographics	Pre-expansion group (2012)	Post-expansion group (2014)	*P*-values^i,j^
Total (n)	77	133	-
Age, median (IQR)^i^	25 (21–28)	22 (20–26)	0.1
Distance (hours), median (IQR)^a,i^	2 (1–2)	2 (1–2)	0.37
Income, median (IQR)^b,i^	1000 (0–5000)	5000 (3000–7000)	<0.01
Ropani, median (IQR)^c,i^	5 (2–12)	5 (2–7)	0.01
Upper caste, n (%)^d,j^	41 (53%)	80 (60%)	0.36
Some literacy, n (%)^e,j^	43 (55%)	114 (86%)	<0.01
Multiparity, n (%)^f,j^	57 (74%)	83 (62%)	0.08
ANC visits Adequate, n (%)^g,j^	53 (69%)	115 (86%)	<0.01
Autonomy, n (%)^h,j^	25 (32%)	65 (49%)	0.02

^a^Distance is defined as the number of hours required to travel from the respondent’s home to the hospital using the fastest mode of transport available to the respondent

^b^Income measured in Nepali Rupees (NRs); regression analysis was done per 1,000 NRs

^c^ *Ropani* is a local measure of farming land in Nepal equal to 508.72 m2

^d^Upper caste is any non-*Dalit* (untouchable) caste

^e^Some literacy is defined as either completion of elementary schooling or any self-reported ability to read in Nepali or English

^f^Multiparity is the number of respondents who had more than one previous birth

^g^Adequacy of ANC visits is defined in accordance with the Nepali government’s minimum of four visits

^h^Women who reported themselves as either the primary or the joint decision-maker were coded as “Autonomous” compared to women who reported their husbands, fathers or mothers-in-law as the primary decision-makers

^i^ *P*-values for non-normal continuous variables were calculated using Wilcoxon rank-sum test

^j^ *P*-values for categorical variables were calculated using Fisher’s exact test

**Table 2:** Factors of institutional births, compared across time and between birth location in each time period

Institutional Birth Factors	2012 n (% of yes respondents)			2014 n (% of yes respondents)			Total n (% all respondents)		
	Home (*n* = 54)	Facility (*n* = 23)	*P*-values^a^	Home (*n* = 31)	Facility (*n* = 102)	*P*-values^a^	2012 (*n* = 77)	2014 (*n* = 133)	*P*-values^a^
Hospital is safer	47 (61.0)	22 (28.6)	0.42	27 (20.9)	102 (79.1)	<0.01	69 (89.6)	129 (97.0)	0.03
Priority on safety	6 (23.1)	20 (76.9)	<0.01	18 (22.0)	64 (78.0)	0.68	26 (33.8)	82 (61.7)	0.01
Priority on cost	2 (100)	0 (0)	>0.99	5 (12.5)	35 (87.5)	0.07	2 (2.6)	40 (30.1)	<0.01
Priority on distance	32 (91.4)	3 (8.6)	<0.01	9 (29.0)	24 (72.7)	0.63	35 (45.5)	33 (24.8)	<0.01
Priority on CEmOC	1 (100)	0 (0)	>0.99	4 (8.9)	41 (91.1)	<0.01	1 (1.3)	45 (33.8)	<0.01
Knowledge of CEmOC availability	10 (76.9)	3 (23.1)	>0.99	3 (3.2)	90 (96.8)	<0.01	13 (20.3)	93 (86.9)	<0.01

^a^ *P*-values are calculated from Fisher’s exact tests

To qualitatively assess the impact of CEmOC expansion on institutional birth, we posed a single open-ended question to each participant: “Tell me the story of your birth.” The enumerators transcribed responses in shorthand in Nepali and then we translated them into English. Two investigators analyzed responses from the pre- and post-expansion data through immersion crystallization [23]. The social contextual model, which illuminates pathways by which social and contextual factors lead to differing health outcomes or health behaviors, informed the analysis [24]. Based on the model, we categorized factors as modifying or mediating on individual, interpersonal, organizational, community, or societal levels. The modifying factors were those that affected the outcome independently of the intervention pathway. The mediating factors were on the pathway between the intervention and the outcome. We undertook this analysis separately in 2012 and 2014 and compared the results to explore how birth stories changed with the expansion to CEmOC.

## Results

We surveyed 98 and 133 women pre- and post-expansion, respectively, including 21 women living outside the catchment area who delivered at Bayalpata Hospital. Bayalpata CHWs do not follow women outside the catchment area population and thus similar women who delivered at home were not a part of the sample. To reduce bias, we excluded these 21 out-of-catchment women from the final analysis, resulting in 77 pre-expansion and 133 post-expansion respondents.

In 2012, 21 women reported delivering in the hospital, 2 in a village clinic (lower level facility), and 54 at home. In 2014, 79 women reported delivering in the hospital, 23 in a village clinic, and 31 at home. In order to verify our exhaustive sampling, we retrospectively reviewed hospital records. There were 30 eligible hospital births documented during the months of data collection in 2012 and 85 in 2014. While we do not have a gold-standard comparator for village clinic and home deliveries, these data suggest we achieved 70% survey coverage in the pre-expansion and 93% survey coverage in the post-expansion hospital delivery groups.

Monthly income, literacy, antenatal visit completion, and autonomy in delivery-care decisions were all significantly higher for the post-expansion group, but the groups were similar in terms of age, distance to facility, median land ownership, parity, and caste as shown in Table 1.

**Fig. 1:**
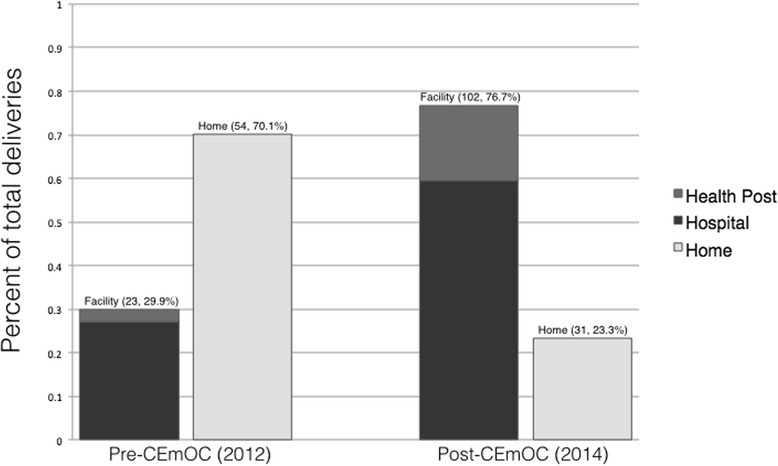
Proportion of facility (including health post and hospital births) and home births before and after roll-out of Comprehensive Emergency Obstetric Care at Bayalpata Hospital. Abbreviations: CEmOC, Comprehensive Emergency Obstetric Care

Institutional birth increased significantly after CEmOC implementation, from 30% (CI 21–41%) to 77% (CI 69–83%) at both hospital (27 to 59%) and village clinic levels (3 to 17%) as shown in Fig. 1.

We report the results of the bivariate analysis of significant factors in Table 2. The number of women who believed the hospital is the safest delivery location and who prioritized safety in decision-making increased post-expansion. Prioritization of distance decreased, while prioritization of cost increased post-expansion. Median travel cost to the facility marginally decreased (300 to 260 NRs, *p* < 0.01) and slightly more women received the government-sponsored incentive payment (91% versus 100%, *p* = 0.03). Finally, post-expansion, more women reported prior knowledge of service availability and prioritization of services.

The logistic regression indicated exposure to CEmOC availability, the belief that the hospital is the safest delivery location, safety prioritization in decision-making, and higher monthly income predict institutional birth (AUC = 0.83). There was a significant interaction of safety prioritization and time, such that pre-expansion women who prioritized safety were seven times more likely to deliver in an institution than those who did not, as shown in Table 3.

**Table 3:** Results of a logistic regression model for institutional birth

Regression term	Estimate	Std. error	Odds ratio	95% CI	*P*-value
CEmOC availability	0.86	0.24	5.6	2.2–15	0.01
Income (per 1000 NRs)	0.07	0.03	1.1	1.0–1.1	0.01
Hospital Safety	1.9	0.69	45	4.8–1300	<0.01
Safety priority	1.02	0.24	7.7	3.2–21	<0.01
CEmOC availability-Safety priority^a^	−1.06	0.24	0.1		<0.01
Safety priority pre-intervention	1.02		7.7		
Safety priority post-intervention	−0.04		0.9		

^a^Interaction term represents the effect of reporting safety as a priority on the likelihood of institutional birth in each time period

Satisfaction with birth experiences increased (87 to 99%, *p* < 0.01). Women delivering in an institution post-expansion were more likely to be satisfied with their delivery care (OR 13, *p* = 0.04). There was no significant difference in perceived adequacy of staff, supplies, or facilities in the pre- and post-expansion institutional birth groups.

In comparing the pre- and post-expansion birth stories, there were several notable differences as demonstrated in Table 4. Mediating factors on an individual and interpersonal level, such as perceptions of safety, knowledge of services, and the awareness of a potential need for services were increasingly common post-expansion. Some explained motivation for hospital births in the context of prior or current birth complications, demonstrating a perceived risk of home birth that could be addressed in the hospital due to CEmOC. One woman noted, “I knew that this hospital provided a complete set of services, just like other hospitals.” Women who gave birth in the village clinic often did so as a secondary option, but largely reported positive impressions of safety and quality.

**Table 4:** Examples of women’s birth stories from 2012 and 2014

	2012	2014
Home	“All day I worked on the farm. At 7 pm, labor pain started. At 12 am, female baby was born at home.” -- “I didn’t know about the hospital and I don’t have anyone who could carry me to the hospital.” -- “I planned to go to the hospital to give birth. I knew about the 1000 NRs [government incentive]. My home condition is very bad and I have no support for people to bring me to the hospital. I had a long course of labor pain, but couldn’t find anyone to carry me to the hospital, so I delivered at home.”	“I had 4 ANC checks - one at hospital and rest 3 at the HP. I had planned to deliver at the HP, but my labor started suddenly and by the time people had gathered to take me to the HP, I had already delivered. I am planning to deliver my next baby at the hospital though.” -- “I had planned to deliver at the hospital and I was on my way as well. But I delivered mid-way. There wasn’t any safe birth kit, no clean cloths. So, it was very difficult.”
Facility	“I started having labor pain and since the hospital is nearby I walked to the hospital and had my baby safely delivered.” -- “A mother of two wanted to give birth in the hospital because they were close by and because the mother thought it would be safer. Her previous children were born at home. Belly pain started late into the night and the family called the ambulance. Unfortunately, the ambulance was not working so it was not able to pick her up. The family got together and found a stretcher. She was carried on a stretcher during the night, one hour away from the hospital. She delivered in the early morning at Bayalpata Hospital.” [as translated]	“I wanted to go to BH to deliver, but there was nobody to help me to the hospital [so I went to the village clinic instead]. My husband is in India and there is just an old mother-in-law at home. But I did complete all four ANCs, took my iron tabs regularly and also the Immunization.” -- “This was my first pregnancy… I completed 4 ANCs. I had also arranged for money and cloths. When the labour pain started, we called the jeep. I delivered normally in the hospital. I am very happy.” -- “I had thought that if I can’t deliver normally then I would deliver via operation. But I could deliver normally, so I am very happy. I also got very good service in the hospital. Because I didn’t have enough money I couldn’t afford to travel to the hospital. But here I was given [government incentive] money for return travel.”

*Abbreviations*: *ANC* Antenatal care, *HP* Health post (local term for village clinic), *NRs* Nepalese Rupees, *BH* Bayalpata Hospital

We identified referrals, birth planning, and preparedness as organizational- and societal-level factors driving institutional birth. One woman said, “per the suggestion of the [CHW]… I also decided to deliver with skilled healthcare personnel.” More women in the post-expansion group mentioned detailed birth planning involving the hospital or village clinic. As one woman described, “I completed four antenatal care visits. I had also arranged for money and clothes. When the labour pain started, we called the jeep [for the hospital].” Pre-expansion, very few women expressed similar birth plans. There were three referrals in the pre-expansion group from the hospital to higher-level facilities. All led to non-institutional births because of difficulties and delays with transportation. After CEmOC implementation, there were no similar referrals. Five women had cesarean deliveries and one woman received a blood transfusion.

**Fig. 2:**
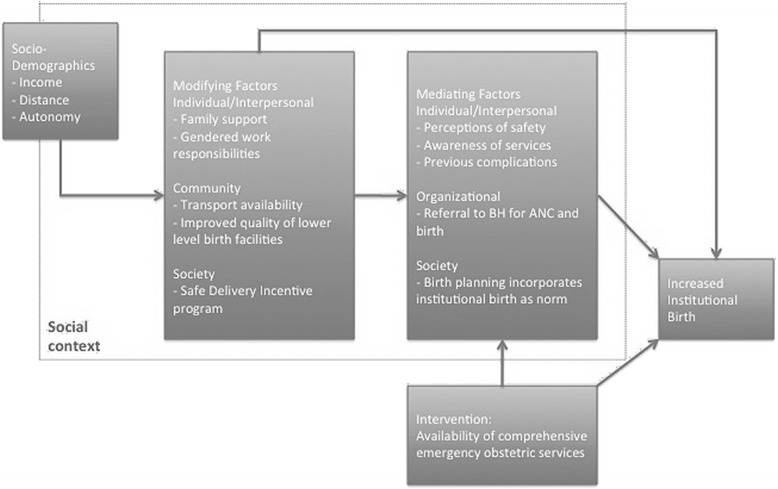
A social contextual theory of change based on qualitative analysis of women’s birth stories. The diagram shows the interplay between modifying and mediating factors, socio-demographic factors, and the intervention. Abbreviations: BH, Bayalpata Hospital; ANC, antenatal care

Modifying factors at the individual and interpersonal level, as shown in Fig. 2, included family (particularly mother-in-law) and partner support, access to financial resources, means of transport to an institutional setting, and gendered work responsibilities. Many women relayed the importance of family and partner support; its absence highlighted the lack of autonomy that many experience. In both years, women who failed to have an institutional birth reported challenges finding travel assistance, often due to inadequate family or community support and planning. Nonetheless, the government-provided financial incentive for travel was a societal-level modifying factor that motivated women to have an institutional birth in both time periods.

## Discussion

The IBR at both the hospital and village clinics more than doubled after CEmOC expansion. To understand this dramatic change, we used logistic regression to assess the importance of CEmOC availability, beliefs about hospital safety, factors reported as important to women’s decisions on delivery location, and knowledge of available services. We also assessed significant changes in income, land ownership, literacy, antenatal care coverage, and locus of decision-making power (self vs. other).

The final regression model indicated that institutional birth was associated with CEmOC service availability, belief in hospital safety, increased income, and prioritization of safety. The overall increase in proportion of women who prioritize safety post-expansion (in both institution and home birth groups) suggests that decision-making factors are sensitive to changes in emergency obstetric services, however prioritization of safety was only significant between institution and home birth groups in the pre-CEmOC period. Awareness of services also increased, though it did not meet significance criteria for inclusion in the final model. These trends suggest an increase in the perceived value of institutional birth driven by improved access to CEmOC.

The substantial increase in institutional birth seen in our quantitative analysis can be partly understood by the mediating social and contextual factors identified in our qualitative analysis. An increased perception of the hospital as a safer and more desirable place to give birth was notable in post-expansion birth stories. Village clinic deliveries were less desirable alternatives to hospital deliveries but were often more achievable due to travel constraints and viewed favorably by women who experienced them. This effect suggests broader normalization of institutional delivery and greater trust in the healthcare system. Normalization of institutional birth may explain why perception of hospital safety strongly predicts institutional birth in both time periods while prioritization of safety only did so prior to CEmOC expansion.

The modifying social and contextual factors identified are those not related to service expansion. The importance of increased income in the quantitative analysis suggests economic development is a modifying factor. In the qualitative analysis, however, we found social support and autonomy more important in both time periods, especially in the context of the pre-existing government incentive. Increasing social support for institutional birth, advancing gender equality, further developing transportation resources, and encouraging birth preparedness should be key targets of future interventions to encourage institutional birth and decrease maternal mortality in this setting.

There are several limitations to our study that encourage caution in generalizing conclusions about the effect of implementing CEmOC on institutional birth in rural areas. It is important to note, given the broad acceptance that cesarean deliveries and blood transfusions save lives, that a randomized, controlled trial would be unethical. As such, questions must be answered via non-randomized approaches. Given the observational pre/post design, the effect of prior trends is not accounted for in the analysis. Increased income may represent a secular “development” trend not fully captured by socio-demographic data. Village clinic quality improvements were not assessed and remain possible confounders given the increase in births at those facilities. We attempted to address these issues through qualitative analysis of individual birth stories.

While we aimed to reach all eligible postpartum women, the sampling technique introduces possible bias because women who delivered at home may have been more difficult to identify or reach by CHWs and we have no gold-standard comparator to validate our coverage. However, our total sample is approximately 70 and 98% of the 110 and 135 deliveries (in 2012 and 2014, respectively) expected by Nepal’s 2013 crude birth rates. These estimates suggest our 70 and 93% coverage of hospital deliveries is unlikely to have systematically under-sampled home deliveries. Our IBRs correlate well with the 35% IBR reported for the region in Nepal’s 2011 Demographic and Health Survey [25], and the 83% IBR reported in a 2014 census conducted in the hospital’s catchment area population [26]. The use of CHWs and nurse midwives as enumerators also increases bias in self-reported perceptions of and preferences for institutional birth. We assume that sampling and self-report biases would be equal across time periods and thus less likely to affect questions of change in institutional birth.

## Conclusion

While demand-generating activities have proven critical to increased IBRs [13], we demonstrate that CEmOC expansion can drive significant demand for institutional births in an impoverished community with previously low access. After CEmOC expansion, women appeared to perceive more benefits of institutional birth and incorporate it into a normative framework that encouraged planning for the extra costs and contingencies required to achieve it. This effect also cascaded down to BEmOC village clinics, with women exhibiting greater trust in the healthcare system overall.

We believe that the demand-generating capacity of CEmOC services should thus be taken into account when considering allocation of maternal and neonatal healthcare resources. These findings support greater expansion of CEmOC services in rural underserved areas even when IBRs are low, as the services are likely to increase both utilization and safety. By increasing demand for institutional births while also making those births safer, surgical obstetric expansion likely has a greater impact on childbirth-related mortality than demand-generating or BEmOC expansion approaches alone.

## Abbreviations

ANC: Antenatal Care
AUC: Area Under Curve
BEmOC: Basic Emergency Obstetric Care
BH: Bayalpata Hospital
CEmOC: Comprehensive Emergency Obstetric Care
CHW: Community Health Worker
CI: Confidence Interval
FCHV: Female Community Health Volunteer
IBR: Institutional Birth Rate
IQR: Interquartile Range
NRs: Nepalese Rupees
OR: Odds Ratio
USD: United States Dollars
